# Multilayer perceptron architecture optimization using parallel computing techniques

**DOI:** 10.1371/journal.pone.0189369

**Published:** 2017-12-13

**Authors:** Wilson Castro, Jimy Oblitas, Roberto Santa-Cruz, Himer Avila-George

**Affiliations:** 1 Facultad de Ingeniería, Universidad Privada del Norte, Cajamarca, Peru; 2 Centro de Investigaciones e Innovaciones de la Agroindustria Peruana, Amazonas, Peru; 3 Facultad de Ingeniería de Sistemas y Mecánica Eléctrica, Universidad Nacional Toribio Rodríguez de Mendoza, Chachapoyas, Peru; 4 Escuela de Doctorado, Departamento de Tecnología de Alimentos, Universidad de Lleida, Lleida, Spain; 5 Unidad de Transferencia Tecnológica Tepic, CONACYT-CICESE, Tepic, Nayarit, Mexico; UMR-S1134, INSERM, Université Paris Diderot, INTS, FRANCE

## Abstract

The objective of this research was to develop a methodology for optimizing multilayer-perceptron-type neural networks by evaluating the effects of three neural architecture parameters, namely, number of hidden layers (HL), neurons per hidden layer (NHL), and activation function type (AF), on the sum of squares error (SSE). The data for the study were obtained from quality parameters (physicochemical and microbiological) of milk samples. Architectures or combinations were organized in groups (G1, G2, and G3) generated upon interspersing one, two, and three layers. Within each group, the networks had three neurons in the input layer, six neurons in the output layer, three to twenty-seven NHL, and three AF (tan-sig, log-sig, and linear) types. The number of architectures was determined using three factorial-type experimental designs, which reached 63, 2 187, and 50 049 combinations for G1, G2 and G3, respectively. Using MATLAB 2015a, a logical sequence was designed and implemented for constructing, training, and evaluating multilayer-perceptron-type neural networks using parallel computing techniques. The results show that HL and NHL have a statistically relevant effect on SSE, and from two hidden layers, AF also has a significant effect; thus, both AF and NHL can be evaluated to determine the optimal combination per group. Moreover, in the three study groups, it is observed that there is an inverse relationship between the number of processors and the total optimization time.

## Introduction

In applied research, it is common to encounter situations in which it is necessary to estimate the behavior of a variable as a function of one or many predictor variables. Traditionally, the solution is provided by statistical regression models for prediction problems, discriminant analysis, or logistic regression models [1, 2]. A group of techniques known as artificial intelligence offers other options, including artificial neural networks, genetic algorithms, and fuzzy logic, among others, which are suitable for solving complex problems [3–6].

Artificial neural networks (ANNs), which are non-linear models inspired by the neural architecture of the brain, were developed in an attempt to model the learning capacity of biological neural systems [7]. A typical ANN architecture known as multilayer perceptron (MLP) contains a series of layers, composed of neurons and their connections. An artificial neuron has the ability to calculate the weighted sum of its inputs and then applies an activation function to obtain a signal that will be transmitted to the next neuron.

The development of MLP networks has two main problems: architecture optimization and training. The definition of architecture is a very relevant point because a lack of connections can make the network incapable of solving the problem of insufficient adjustable parameters, whereas an excess of connections may cause an over-fitting of the training data [8]. Consequently, training MLP networks for large datasets is very time consuming [9].

Determination of the optimal architecture is a constant goal in research papers [10–12], which attempt to minimize an objective function, mean squared error or prediction residual sum of squares errors and avoid the oversize of the network; the method used in these research works is trial and error. However, the trial and error method limits the capacity of analyzable architectures and reduces the likelihood of finding an optimal architecture, particularly if we have a large number of possible architectures. Different approaches have been proposed to optimize the architecture of an MLP network, for example, back-propagation [13], genetic algorithms [14], ant colony [15], bee swarm [16], and Tabu search [17], among others.

Similarly, different approaches have been proposed to manage the expensive training phase, for example, the use of multicore CPU [18–20], cloud computing [21] and hybrid algorithms [8, 15], among others.

In this paper, we focus on the problem of constructing an optimal multilayer perceptron network architecture. Given the popularity and easy access to equipment with large multi-core multiprocessing or GPU capabilities, which enable the parallel calculation of multiple operations, a comprehensive approach to finding the optimal architecture of a multilayer perceptron network is proposed. Four versions of the proposed approach, i.e., sequential, multi-core, GPU and a hybrid algorithm, are introduced.

The objectives of this research are as follows: (a) propose a methodology for optimizing multilayer-perceptron-type neural networks, (b) evaluate the effects of the different structural parameters on the sum of squares error, and (c) evaluate the performance of the optimization process using parallel computing techniques.

## Materials and methods

### Materials

**Fig 1:**
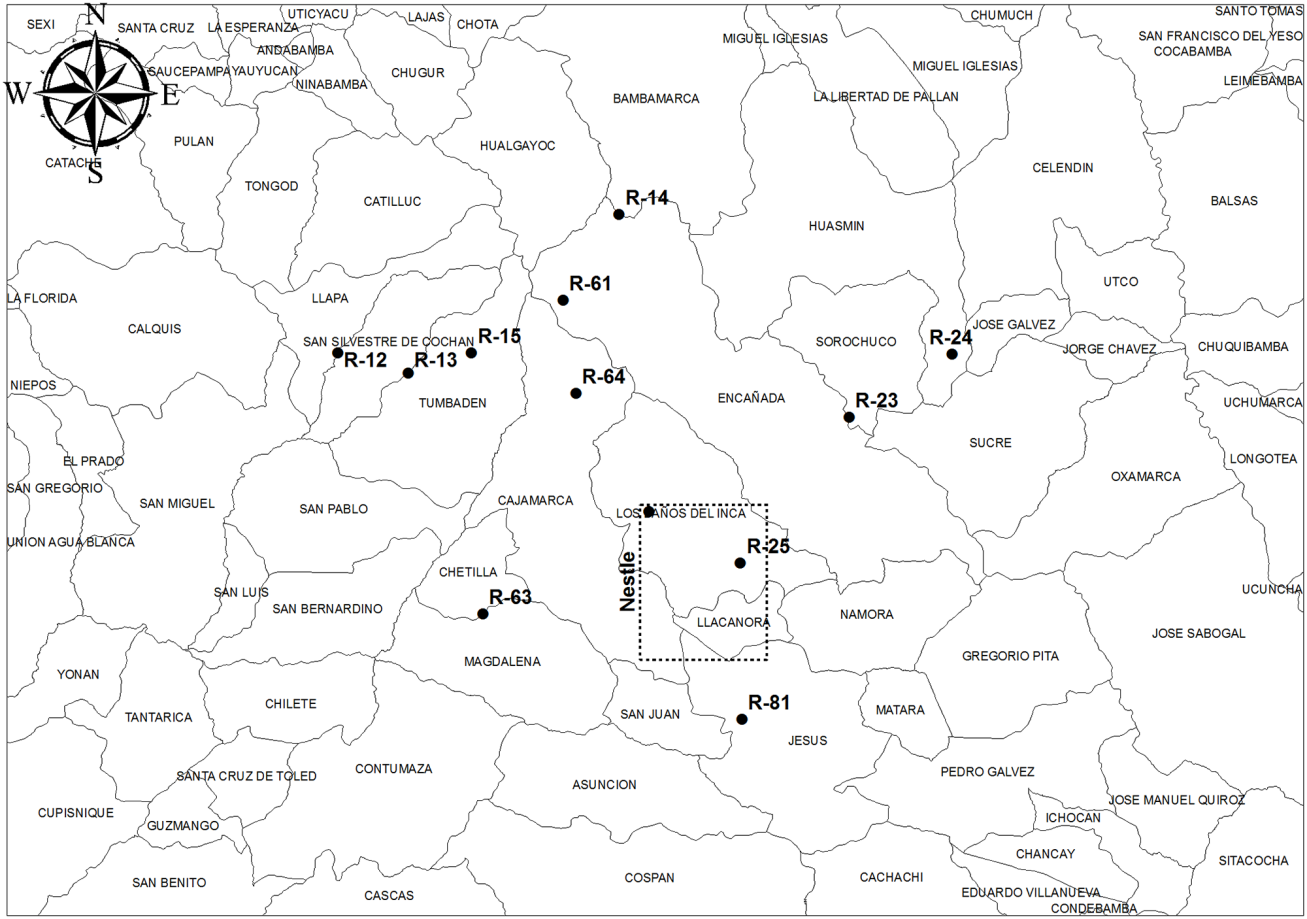
Cajamarca region map.

As a biological material, 252 milk samples from Holstein cows (40 cc/sample) were collected. Sampling was conducted between July and August 2014. The milk samples were not collected exclusively for this work; they were provided by employees of Nestlé S. A. from 12 centers located in the countryside of Cajamarca, Peru. Fig 1 shows the map of the Cajamarca region, where the twelve points of milk collection are indicated, and Table 1 shows the geographic coordinates of each of the twelve points.

**Table 1:** Dataset geographic coordinates.

Point	Latitude	Longitude
R-12	−6°56′28.451″	−78°46′35.976″
R-13	−6°57′37.054″	−78°42′45.183″
R-14	−6°48′59.133″	−78°31′4.870″
R-15	−6°56′30.461″	−78°39′14.912″
R-23	−7°0′12.497″	−78°18′31.890″
R-24	−6°56′46.243″	−78°12′51.233″
R-25	−7°8′9.444″	−78°24′33.531″
R-61	−6°53′40.768″	−78°34′12.428″
R-63	−7°10′51.045″	−78°38′44.912″
R-64	−6°58′46.375″	−78°33′31.697″
R-72	−7°5′19.486″	−78°29′33.630″
R-81	−7°16′45.528″	−78°24′33.087″

Nestlé is an internationally recognized company that is very committed to the welfare of animals, as indicated by their strict guidelines for animal care [22].

### Computing system

A computer called Perseo was used for the experiment, which is a shared-memory multiprocessor. Perseo is part of the network of computer equipment belonging to the *Centro Nayarita de Innovación y Transferencia de Tecnología A.C*, Mexico.

The main characteristics of Perseo are as follows:

- Processor = 24 Intel(R) Xeon(R) CPU ES-2670 v3 @ 2.30 GHz
- CPU cores = 24
- RAM Memory = 32 GB
- Graphic card = NVIDIA Quadro K4200
- Operating System = Ubuntu 14.04

The software used for implementing the logical sequences was MATLAB version 2015a.

### Experimental methodology

#### Obtaining training and validation data

Samples were packed in sampling bottles and transported to the lab of the Nestle plant, located in the city of Cajamarca, where they were characterized as shown in Table 2.

**Table 2:** Analyses performed on the milk samples.

	Parameter	Method	Source
Input	Density	Lactodensimeter (AOAC 925.22)	[23]
	Oxidation-Reduction Potential	Reaction time to methylene blue	[24]
	Potential of Hydrogen (pH)	Potentiometer	[25]
Output	Proteins	Infrared spectroscopy (NTP 202.130:1998)	[26]
	Lactose		
	Total solids		
	Solids-fat		
	Solids-non-fat		
	Minerals		

The data obtained from each sample were divided into three input values: density (Dn), oxidation-reduction potential (Rd), and potential of hydrogen (pH). Moreover, six output parameters were defined: proteins (Pr), lactose (Lc), total solids (Ts), solids-fat (Sf), solids-non-fat (Snf), and minerals (Mn).

#### Condensed architecture for multilayer perceptrons

**Fig 2:**
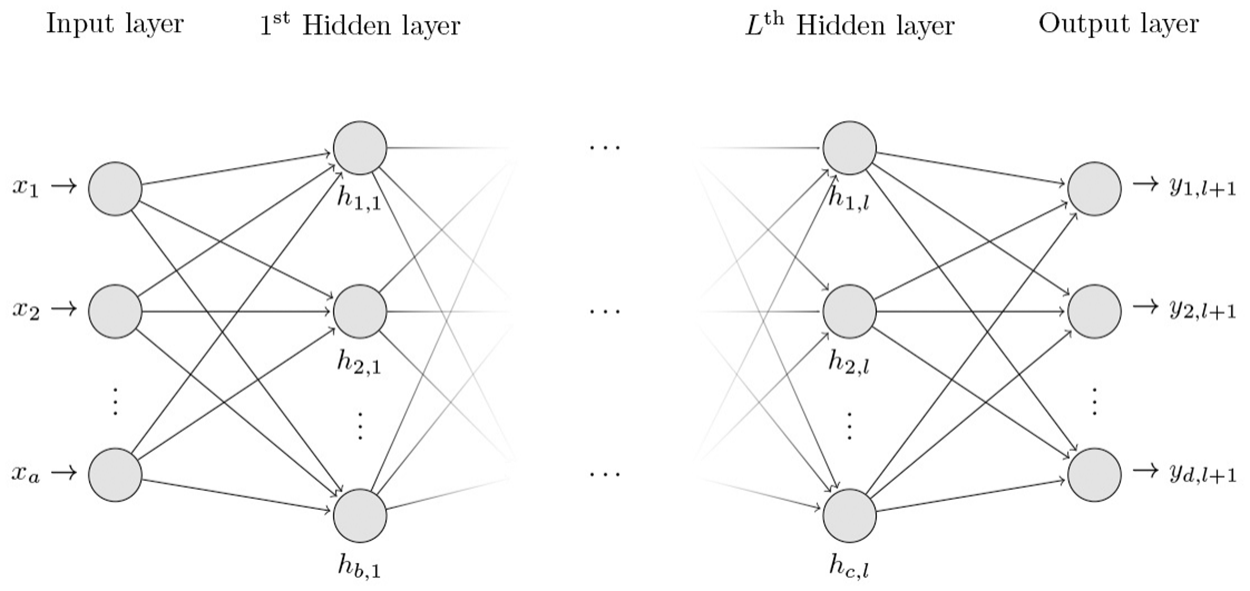
Condensed architecture for multilayer perceptron.

Fig 2 shows the proposed multilayer perceptron architecture, which is based on the following works [27–29].

The structural parameters to evaluate and their ranges were established in accordance with [30]; see Table 3.

**Table 3:** Ranges in structural parameters.

Parameters	Range
Input neuron layer (IN)	3
Output neuron layer (ON)	6
Number of hidden layers (HL)	[1-3]
Neurons per hidden layer (NHL)	[3-27]
Activation functions^⋆^ (AF)	[1-3]

^⋆^ (1) Hyperbolic tangent sigmoid (tan-sig)

(2) Log sigmoid (log-sig)

(3) Linear

**Fig 3:**
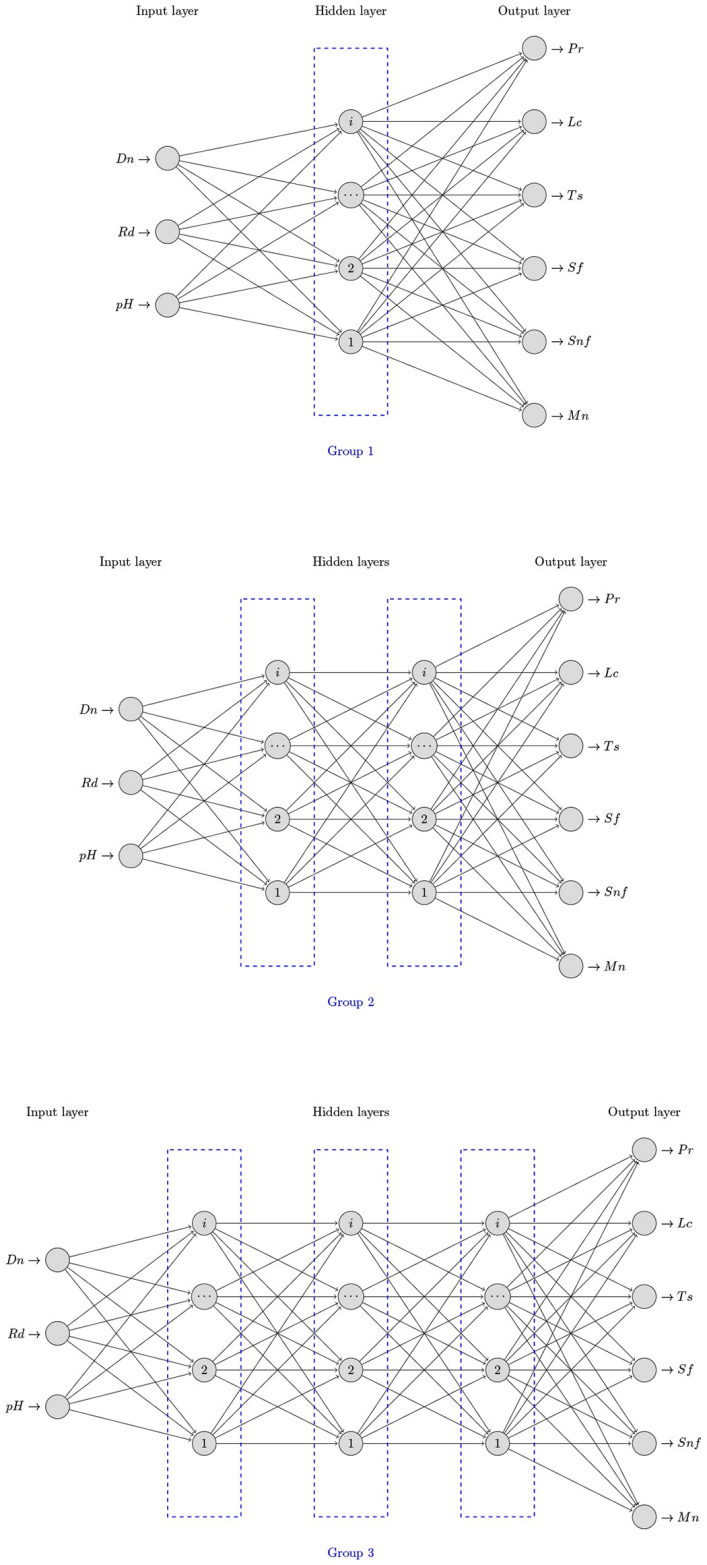
Groups of neural architectures proposed for the study.

After modifying the number of hidden layers, inter-spacing from one to three hidden layers, different architectural groups were generated, as shown in Fig 3.

For the bias parameter values and weightings, prior initiation to zero was determined during the optimization process.

#### Experimental designs and generation of combinations per group

Table 4 shows the experimental designs used in this research work; the architectures were generated per group and evaluated using factorial designs without repetition.

**Table 4:** Experimental designs used in this research.

Group	Designs	Factors	
		Name	Levels
1	1HL2AF	NHL1	[3 6 9 12 15 18 21 24 27]
		AF1	[1 2 3]
		AF2	[1 2 3]
2	2HL3AF	NHL1	[3 6 9 12 15 18 21 24 27]
		NHL2	[3 6 9 12 15 18 21 24 27]
		AF1	[1 2 3]
		AF2	[1 2 3]
		AF3	[1 2 3]
3	3HL4AF	NHL1	[3 6 9 12 15 18 21 24 27]
		NHL2	[3 6 9 12 15 18 21 24 27]
		NHL3	[3 6 9 12 15 18 21 24 27]
		AF1	[1 2 3]
		AF2	[1 2 3]
		AF3	[1 2 3]
		AF4	[1 2 3]

The position of each element within the groups and the number of combinations (treatments) per experimental design are detailed in Table 5.

**Table 5:** Treatments per experimental design.

Design	Distribution of elements^⋆^	Number of Treatments
1HL2AF	IN, NHL1_*i*_, ON, AF1_*l*_, AF2_*m*_	63
2HL3AF	IN, NHL1_*i*_, NHL2_*j*_, ON, AF1_*l*_, AF2_*m*_, AF3_*n*_	2 187
3HL4AF	IN, NHL1_*i*_, NHL2_*j*_, NHL3_*k*_, ON, AF1_*l*_, AF2_*m*_, AF3_*n*_, AF4_*o*_	59 049

^⋆^Sub-indexes correspond to the levels assumed in each combination.

Combinations per group according to the proposed designs were generated using the statistical software Statgraphics Centurion XVI.

#### Perceptrons per group of combinations: Generation and evaluation

To create, train and simulate MLP-type networks, MATLAB’s Neural Network Toolbox was used, particularly the function newff, whose syntax is shown in Eq (1).

$$\begin{array}{c} net=\text{newff}(p,t,G(i,j)) \end{array}$$ (1)

where:

*p*: is the vector of input values.
*t*: is the vector of output values.
*G*_*i*,*j*_ is a combination per group (*i*: group; *j*: combination number).

**Fig 4:**
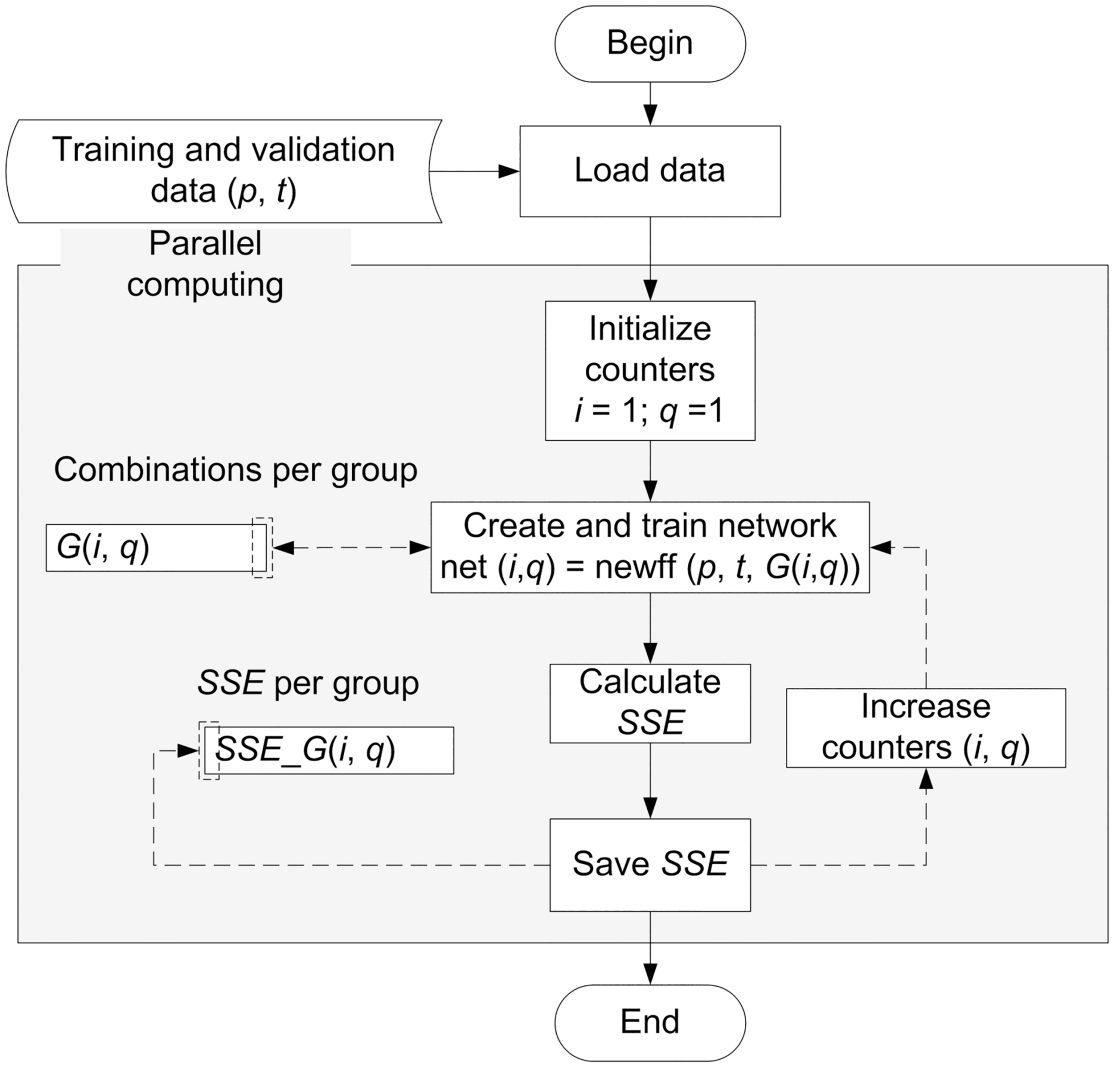
Sequence for constructing, training, and evaluating networks.

The networks per group were evaluated by determining the sum of squares error (SSE) using a logical sequence. This sequence was implemented in the mathematical software MATLAB 2015a; see Fig 4.

Likewise, due to the large number of combinations per group and the high calculation time cost, the analysis sequence was implemented using profiling, vectorization and parallel computing techniques.

Initially, a sequential version of the proposed algorithm was developed (MLP-SEQ), which was implemented using profiling and vectorization techniques. However, since the algorithm has a comprehensive search approach, it is time consuming. Theoretically, the time required by an algorithm to calculate the solution to a given problem using a single processor could be linearly decreased by adding more processors. Following this idea, we developed the following algorithms to attempt to reduce the processing time.

- NNTB-GPU development is based on MATLAB’s Neural Network Toolbox, where the idea is to take advantage of the computation based on the GPU and to process the search space in parallel.
- NNTB-CPU is also based on MATLAB’s Neural Network Toolbox, where the idea is to take advantage of multi-CPU architectures to process data faster.
- NNTB-Hybrid merges the two previous approaches.
- PCTB-CPU is based on MATLAB’s Parallel Computing Toolbox, proposing a distributed computing approach (master-worker).

#### Analysis of evaluation times per group of combinations

Acceleration and efficiency are some of the most important measurements for assessing the quality of the implementation of a logical sequence (algorithm) on an architecture of multiprocessors [31]. The acceleration of a logical sequence implemented in parallel executed using *n* processors is the ratio between the time that it takes the best logical sequence implemented sequentially to be executed using a single processor in a computer and the time that it takes the corresponding logical sequence implemented in parallel to be executed on the same computer using *n* processors; see Eq (2).

$$\begin{array}{c} S=\frac{T_{o}}{T_{n}} \end{array}$$ (2)

where

*S*: Acceleration.
*T*_0_: Computing time with one processor.
*T*_*n*_: Time with *n* processors.

If acceleration is normalized by dividing it by the number of processors, then efficiency is obtained; see Eq (3).

$$\begin{array}{c} \eta =\frac{S_{n}}{n} \end{array}$$ (3)

where

*η*: Efficiency.
*S*_*n*_: Speedup with one processor.
*n*: Number of processors.

Parallel and sequential versions of the logical sequence shown in Fig 4 were developed. The obtained results were analyzed in terms of acceleration and efficiency.

## Results and discussion

### Training data

The data collected during the milk analysis stage and later used in constructing, training, and evaluating the networks are shown in Table 6.

**Table 6:** Training and validation data per neural network.

	Variables	Units	Values			
			min	max	$\bar{x}$	*σ*
Input	Density (Dn)	g/ml	1.026	1.03	1.028	0.001
	Oxidation-Reduction Potential (Rd)	hours	6.5	6.79	6.63	0.049
	Potential of Hydrogen	—	6	8	6.5	0.637
Output	Proteins (Pr)	g/100 ml	2.69	3.33	3.005	0.14
	Lactose (Lc)	g/100 ml	4.31	5.24	4.85	0.187
	Solids total (St)	g/100 ml	10.89	13.14	12.22	0.433
	Solids-fat (Sf)	g/100 ml	3	4.1	3.62	0.183
	Solids-non-fat (Snf)	g/100 ml	7.73	9.27	8.54	0.31
	Minerals (Mn)	g/100 ml	0.41	0.71	0.7	0.023

The results shown are similar to those reported by [32], who analyzed the microbiological composition and quality of dairy cattle in southern Peru.

### Combinations by groups of neural architectures

**Fig 5:**
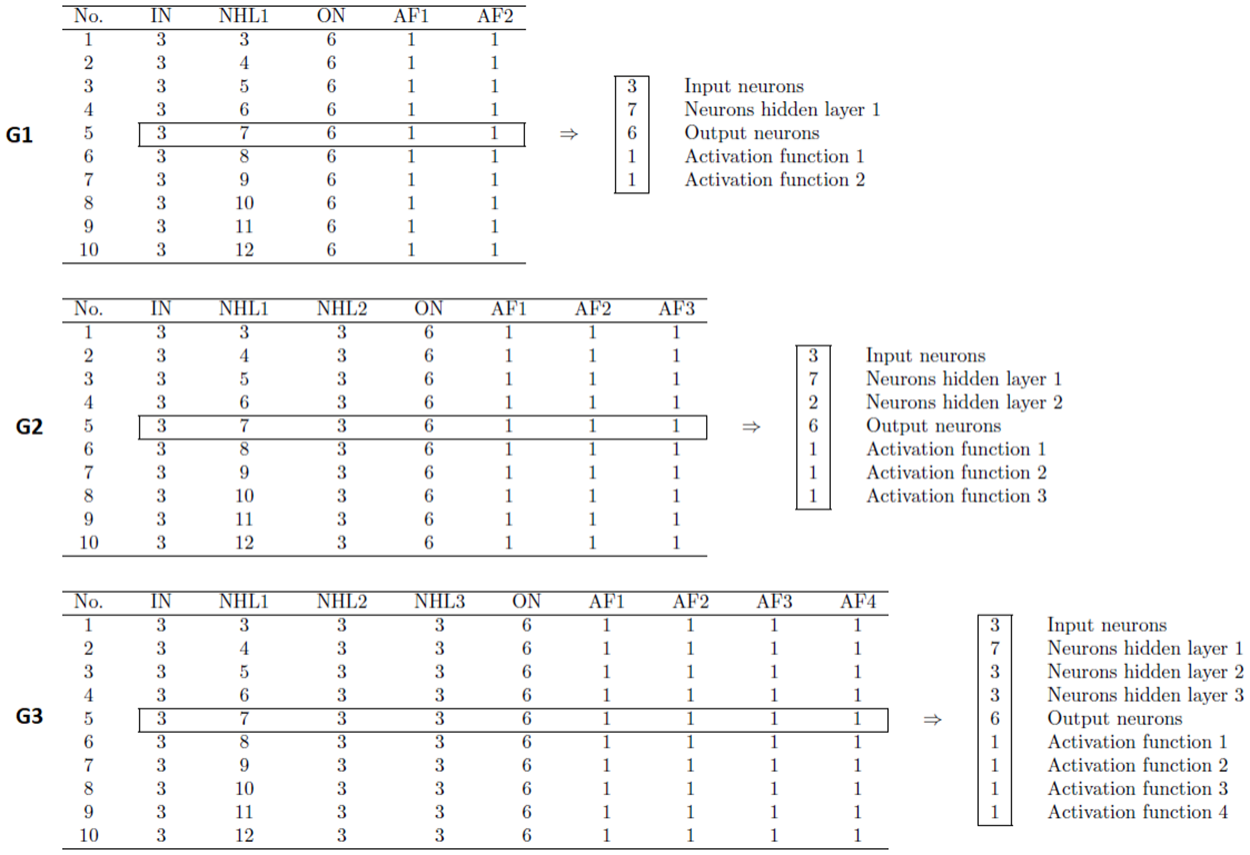
First ten combinations for G1, G2, and G3 and interpretation.

The first ten combinations by groups of neural architectures, which were used in the construction of neural networks, are shown in Fig 5.

As can be appreciated, the architecture groups (G1, G2, and G3) differ in the number of hidden layers (HL), which is a parameter that controls network accuracy. Within each group, the differences are found in the number of neurons and the type of activation function. This parameter has been researched as an element for neural network optimization in previous works, such as those reported by [33, 34].

### Architecture group analysis

The idea of applying parallel computing techniques in the area of neural networks has been used in the following research papers [18–21]. As shown, the use of distributed computing has been used mainly in the training phase of the neural network, which generally consumes considerable computational resources when neural networks are vast and complex.

In this work, we developed three algorithms using the parallel techniques provided in MATLAB’s Neural Network Toolbox; these algorithms are NNTB-GPU, NNTB-CPU, and NNTB-Hybrid. To compare their performance, we used the G2 dataset. The NNTB-GPU algorithm used all the resources provided by a NVIDIA Quadro K4200 card; NNTB-CPU was run using four processors; finally, NNTB-Hybrid was run using four processors and the graphics card. The execution time, in seconds, of each algorithm was 3 028, 2 911, and 3 634, respectively, with the algorithm NNTB-CPU obtaining better performance.

However, by running our sequential algorithm using the G2 dataset, the processing lasted only 637 seconds. The reasons for this result could be the following: (1) the functions that we use to parallelize the NNTB-GPU, NNTB-CPU and NNTB-Hybrid algorithms obtain good performance when neural networks are complex, i.e., many input and output neurons, many hidden layers, and so forth. However, as shown in Fig 3, the neural networks that are processed in this research work do not typically have a complex structure. For this reason, the three approaches previously mentioned spend more time in establishing the parallel environment than in processing a particular neural network. In fact, the problem that we are facing is to process many small neural networks to find the optimal architecture.

Therefore, we developed the PCTB-CPU algorithm, which is based on the general functions of the MATLAB PCTB. This algorithm uses a master-slave approach and creates a balanced distribution of work among all available workers, i.e., the total number of architectures to be tested is divided equally among the workers. The workers report their partial results to the master, and the master is responsible for integrating all the information and submitting the result. Following the previous example, the PCTB-CPU algorithm was tested using four workers to process the dataset G2. This time, the duration was 218 seconds, which means that it achieved 85% of the theoretical acceleration. Using this algorithm, the datasets G1, G2, and G3 were processed.

**Fig 6:**
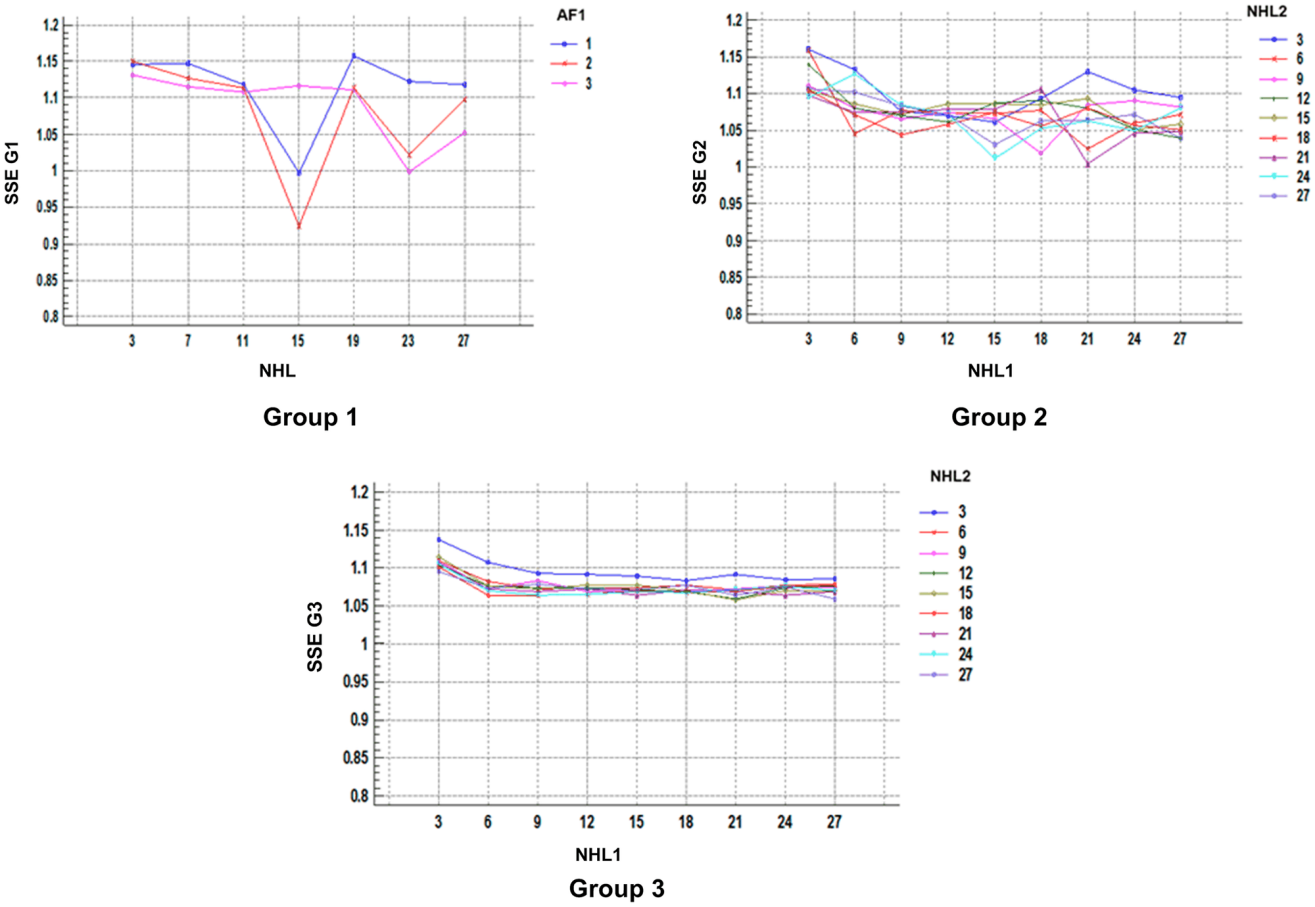
SSE for each group.

Regarding the resulting SSE by groups, according to factors, they are illustrated in Fig 6, and they show that increasing the hidden layers reduces SSE dispersion, generating more robust multilayer perceptrons; the results agree with the work of Garcia *et al.* [35] about the relationship between the number of layers and the network efficiency, as well as with the results obtained by Izquierdo *et al.* [33], who evaluated different structures until they determined the optimal ones for their study conditions.

The analysis of the multifactorial variance for the SSE within each group according to NHL and AF is shown in Tables 7–9.

**Table 7:** Variance analysis^⋆^ for SSE G1.

Source	Sum of squares	Degrees of freedom	Mean square	Ratio-F	Value-P
Main Effects					
NHL1	0.1243	6	0.0207	4.86	0.0005
AF1	0.0145	2	0.0073	1.7	0.1927
AF2	0.0247	2	0.0124	2.9	0.0640
Residual	0.2217	52	0.0043		
Total (corrected)	0.3853	62			

^⋆^ Reliability level 99%.

**Table 8:** Variance analysis^⋆^ for SSE G2.

Source	Sum of squares	Degrees of freedom	Mean square	Ratio-F	Value-P
Main Effects					
NHL1	0.6434	8	0.0804	13.41	0
NHL2	0.2346	8	0.0293	4.89	0
AF1	0.9168	2	0.4584	76.45	0
AF2	0.1084	2	0.0542	9.04	0.0001
AF3	0.2903	2	0.1451	24.2	0
Residual	12.9758	2164	0.006		
Total (corrected)	15.1693	2186			

^⋆^ Reliability level 99%.

**Table 9:** Variance analysis^⋆^ for SSE G3.

Source	Sum of squares	Degrees of freedom	Mean square	Ratio-F	Value-P
Main Effects					
NHL1	7.6283	8	0.9535	147.58	0
NHL2	2.7694	8	0.3462	53.58	0
NHL3	2.464	8	0.308	47.67	0
AF1	38.776	2	19.3881	3000.69	0
AF2	0.4399	2	0.2199	34.04	0
AF3	4.6349	2	2.3174	358.67	0
AF4	3.5627	2	1.7814	275.7	0
Residual	381.3121	59016	0.0065		
Total (corrected)	441.587	59048			

^⋆^ Reliability level 99%.

From the P-value, it is determined that the NHL has a statistically significant effect on the SSE in each group and that as of the second group (second hidden layer), the AF is added to it.

**Fig 7:**
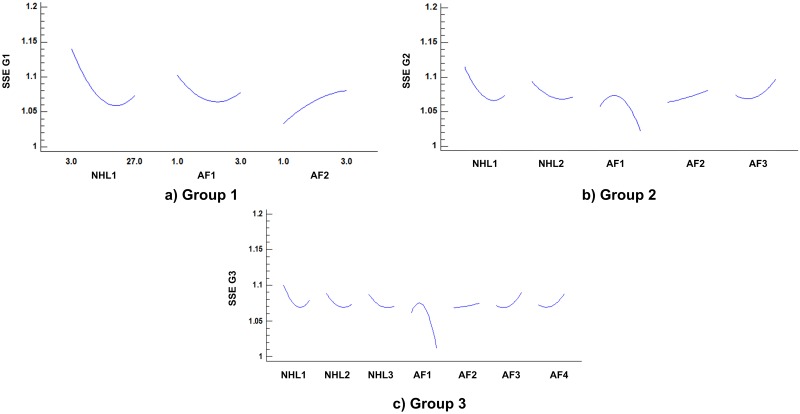
Interaction of structural parameters per group.

When evaluating the SSE and their relationship with the structural parameters per group, images a, b, and c in Fig 7 are obtained. It can be observed that increasing the NHL reduced the SSE until a minimal value is reached, and later increases cause an increase in the SSE. This result is possibly due to the effect of over training, as explained by Velásquez *et al.* [36] in their study.

It is also observed that the SSE exhibits a different behavior for the various types of AFs and the layer to which they connect. Therefore, the neural structure should be optimized by minimizing the SSE according to the NHL and AF, determining the best combination of said parameters. Table 10 presents the optimal values for the evaluated groups, as well as the minimal SSEs.

**Table 10:** Optimal values of NHL and AF per group.

Factor	Optimal values		
	G1	G2	G3
NHL1	22	25	18
NHL2	—	27	27
NHL3	—	—	26
AF1	2	3	3
AF2	1	1	3
AF3	—	2	1
AF4	—	—	1
SSE	1.0217	0.9876	0.9847

### Optimization process times

During the experimental process, to measure the performance of the proposed logical sequence, three parameters (computing time, acceleration and efficiency) were used as references.

**Fig 8:**
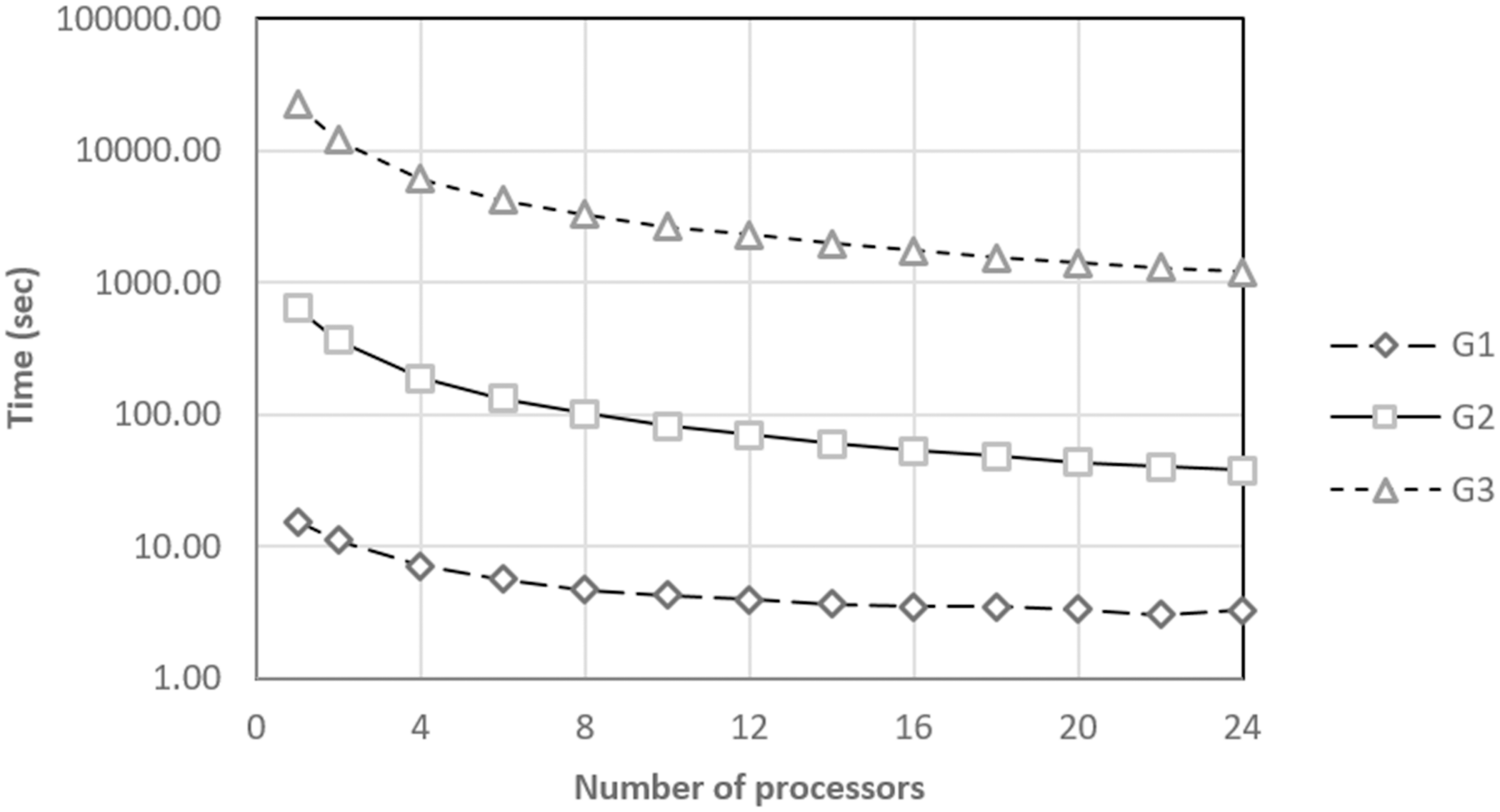
Calculation times per group and number of processors.

The times for determining the SSE in the various groups are shown in Fig 8. According to [33], the processing times may vary according to the characteristics of the equipment where the logical sequence is implemented. However, the trends shown by the results are similar to those obtained in the work by [37] when the number of processors was successively increased to the process.

**Fig 9:**
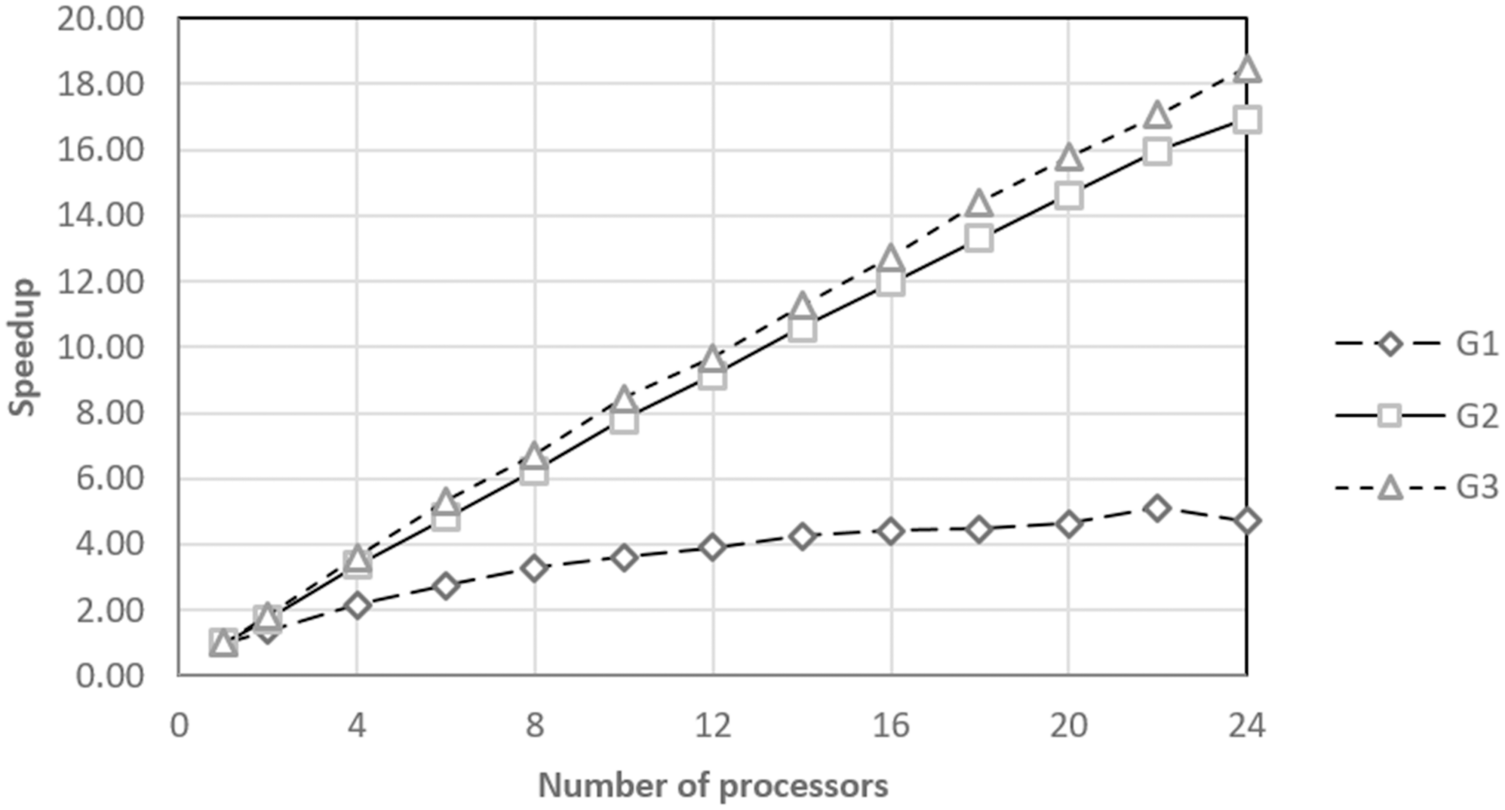
Acceleration of optimization per groups.

From the analysis of the optimization process times, it is inferred that the implementation requires a large calculation capacity for practical applications. In that sense, the speedup values of the optimization process, Fig 9, show that there is an inverse relationship between the number of processors and the total optimization times with a constant tolerance for the various groups under study.

**Fig 10:**
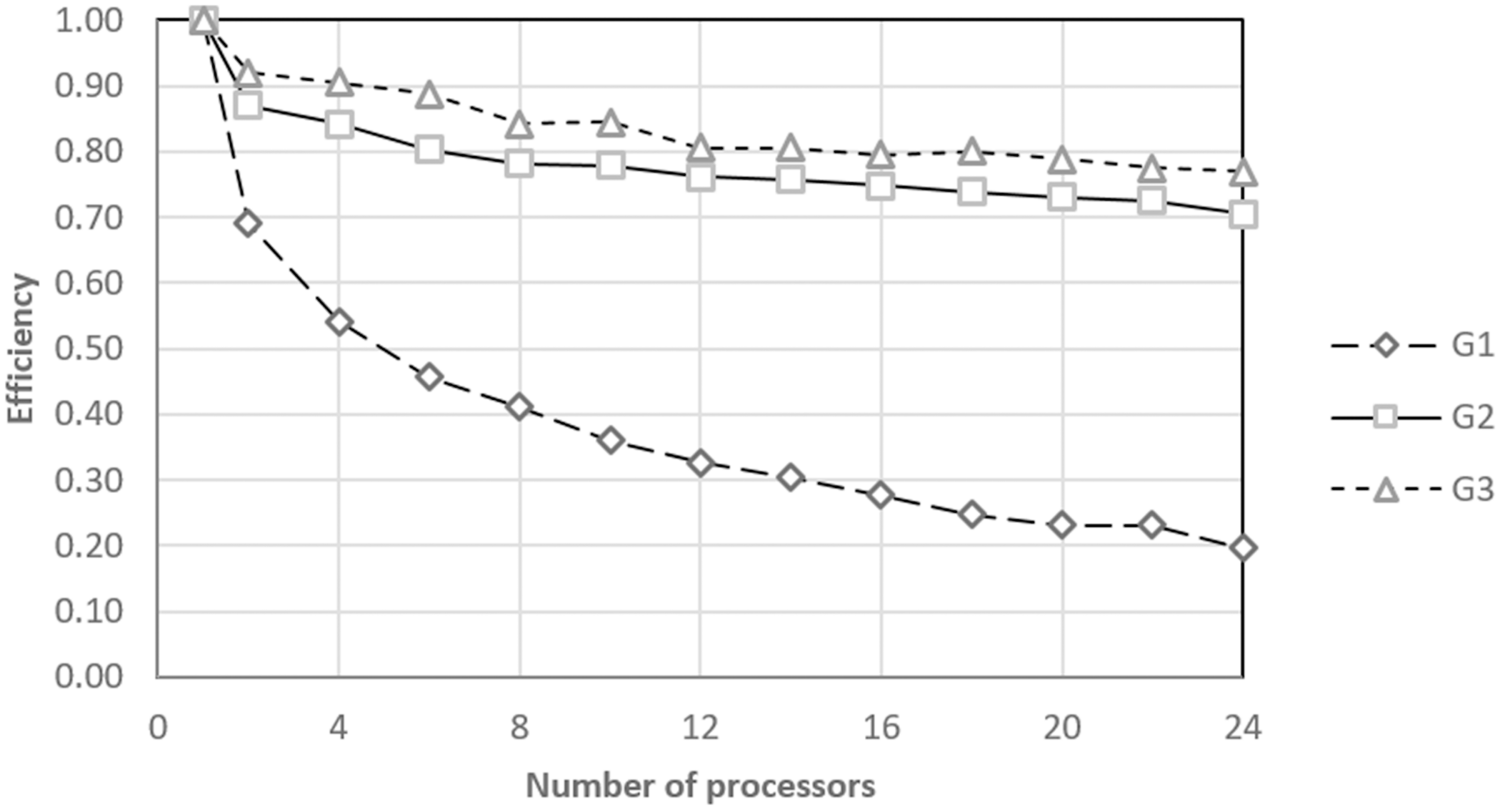
Efficiency in the optimization of the groups.

The information shown in Figs 8 and 9 are complemented with an analysis of the logical sequence efficiency. Fig 10 shows the efficiency achieved by the proposed parallel algorithm each time that it was tested using the G1, G2, and G3 treatment sets. For the G1 case, the efficiency quickly decreases, which may be because more time is spent establishing the parallel environment than processing treatment. However, for the G2 and G3 cases, the algorithm reported an efficiency of over 70% even when using the maximum number of processors. This result indicates that the proposed parallel algorithm could scale considerably for larger experimental designs.

## Conclusions

The optimal architecture of a multilayer-perceptron-type neural network may be achieved using an analysis sequence of structural parameter combinations.

The number of hidden layers and the number of neurons per layer have statistically significant effects on the SSE. Likewise, the SSE shows a different behavior with respect to the various types of AF and the layer to which they connect.

The implementation of the logical sequence of the optimization is possible by applying parallel computing to the process, which reduces the process time and, depending on the number of processors, improves the performance.

## Supporting information

S1 File Input data. (ZIP)
